# Inherited heterozygous Fanconi anemia gene mutations in a therapy-related CMML patient with a rare *NUP98-HOXC11* fusion: A case report

**DOI:** 10.3389/fonc.2022.1036511

**Published:** 2022-10-19

**Authors:** Kefeng Shen, Meilan Zhang, Jiachen Wang, Wei Mu, Jin Wang, Chunyan Wang, Shugang Xing, Zhenya Hong, Min Xiao

**Affiliations:** Department of Hematology, Tongji Hospital, Tongji Medical College, Huazhong University of Science and Technology, Wuhan, China

**Keywords:** Fanconi anemia gene, hereditary cancer predisposition syndromes, ovarian cancer, therapy-related CMML (t-CMML), *NUP98-HOXC11* fusion

## Abstract

Fanconi anemia (FA) genes play critical roles in the repair of DNA lesions. Non-FA (or underlying FA) patients harboring heterozygous germline FA gene mutations may also face an increased risk of developing bone marrow failure, primary immunodeficiency disease, and hereditary cancer predisposition syndromes. We report a female patient who suffered from ovarian cancer at 50 years of age. During the initial treatment, six cycles of docetaxel and carboplatin (DC) combination chemotherapy were administered followed by two cycles of docetaxel maintenance therapy. Then, she received a routine follow-up every 3 months for the next 3 years, and all the results of the examination and laboratory tests were normal. Unfortunately, at 54 years of age, she developed a secondary cancer of therapy-related (t-) chronic myelomonocytic leukemia (t-CMML). After two courses of a highly intensive induction chemotherapy regimen with DAC (decitabine) and HAA (homoharringtonine, cytarabine), the patient suffered from severe and persistent bone marrow failure (BMF). Targeted next-generation sequencing (NGS) of a panel of 80 genes was performed on her initial bone marrow aspirate sample and identified *PTPN11, NRAS*, and *DNMT3A* somatic mutations. In addition, RNA sequencing (RNA-seq) revealed a rare *NUP98-HOXC11* fusion. Whole-exome sequencing (WES) verified *RAD51C, BRIP1, PALB2*, and *FANCG* heterozygous germline mutations of the FA pathway, which were further confirmed in buccal swab samples by Sanger sequencing. For this patient, we hypothesized that an altered FA pathway resulted in genomic instability, hypersensitivity to DNA-crosslinking agents or cytotoxic chemotherapeutics, and unsuccessful DNA damage repair. Consequently, she developed ovarian cancer and secondary t-CMML and then suffered from BMF and delayed post−chemotherapy bone marrow recovery after several chemotherapy courses. This case highlights the importance of genetic counseling in patients with hematopoietic neoplasms with high clinical suspicion for carrying cancer susceptibility gene mutations, which require timely diagnosis and personalized management.

## Introduction

The Fanconi anemia (FA) pathway (also known as the *FA-BRCA* pathway) is involved in the repair of DNA lesions by homologous recombination, which plays a vital role in the maintenance of genomic stability (1). To date, researchers have already identified germline mutations in 22 specific genes associated with the FA pathway, each accounting for an individual FA complementation group (2). Patients with FA gene mutation are hypersensitive to DNA damage and unable to successfully repair damaged DNA when exposed to DNA-crosslinking agents, cytotoxic chemotherapeutics, and ionizing radiation (3, 4). Throughout the lifetime of patients with an FA gene mutation, DNA damage increasing accumulates, which would lead to a complex clinically and genetically heterogeneous disorder characterized by developmental abnormalities, bone marrow failure (BMF), immune deficiency, and a high risk of developing various cancers (e.g., Fanconi anemia, breast/ovarian cancer, leukemia) (5–7).

**Figure 1:**
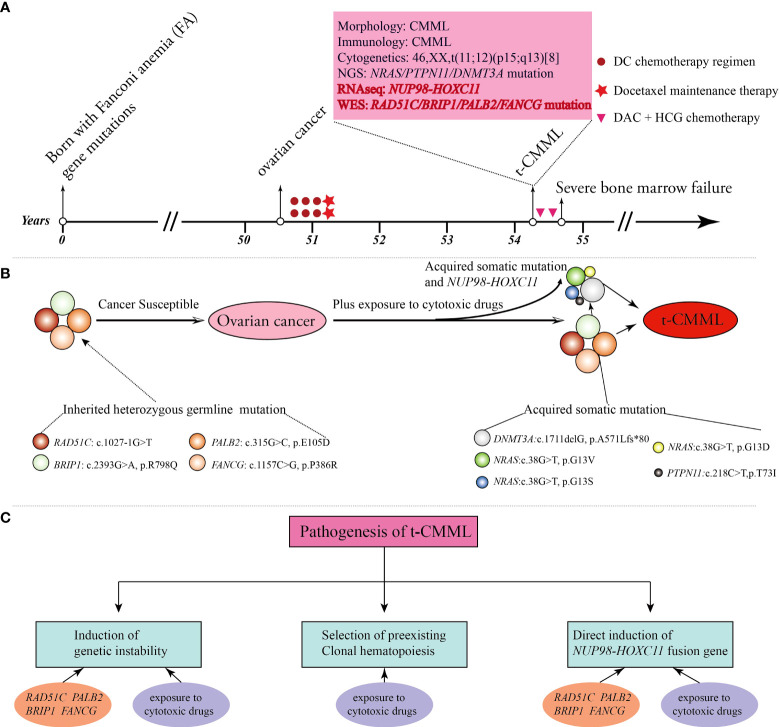
A brief summary of the medical history and comprehensive molecular-genetic analysis. **(A)** Patient timeline of key clinical events and laboratory test results. **(B)** The patient was born with four Fanconi anemia (FA) gene mutations and germline predisposition to cancers. She developed ovarian cancer, and then the altered FA pathway plus exposure to cytotoxic drugs promoted the emergence of acquired somatic mutations and the *NUP98-HOXC11* fusion, all of which contributed to the development of secondary t-CMML. **(C)** Potential biological mechanisms contributing to the pathogenesis of t-CMML.

Here, we present the case of a 54-year-old female patient with multiple FA gene mutations. She was healthy, and with a normal history in her early years. Unfortunately, she developed ovarian cancer, secondary t-CMML, and post−chemotherapy BMF sequentially in her fifties. Comprehensive genetic testing showed that many molecular variations (including FA gene germline mutations, *RAS* and epigenetic pathway somatic mutations, and *NUP98-HOXC11* fusion) were highly linked to her serious and complex medical history (**Figures 1A, B**).

## Case report

**Figure 2:**
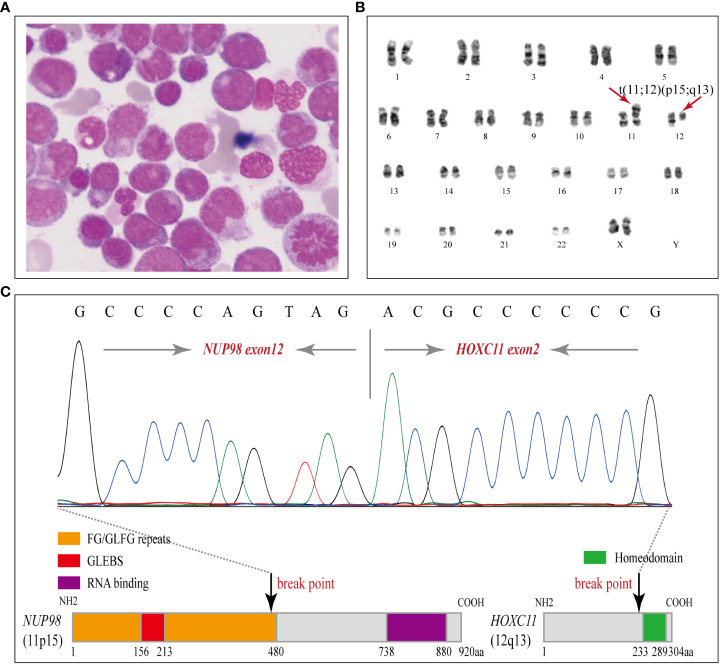
Morphology, karyotyping and molecular characterization of the *NUP98-HOXC11* fusion. **(A)** A diagnostic bone marrow (BM) aspirate smear revealed dysplasia in granulocytic and megakaryocytic cell lineages and enriched naïve monocytes (Wright stained, × 1000). **(B)** Karyotyping performed on the diagnostic BM revealed 46, XX, t(11;12)(p15;q13) [8]. **(C)** Upper panel, Sanger sequencing was performed to verify the *NUP98-HOXC11* fusion transcript containing exon 12 of *NUP98* fused to exon 2 of *HOXC11*. Lower panel, a schematic diagram showing the *NUP98-HOXC11* in-frame fusion and phenylalanine-glycine/glycine-leucine-phenylalanine-glycine (FG/GLFG) repeats and Gle2-binding sequence (GLEBS) domain in the amino-terminal portion of *NUP9*8 retained in the fusion protein.

A 54-year-old female patient was admitted to the department of hematology in our hospital because of scattered ecchymosis on limb skin in July 2020. A complete blood count (CBC) test showed hyperleukocytosis and monocytosis with a white blood cell count (WBC) of 100.1×10^9^/L, monocyte count of 47.1×10^9^/L, hemoglobin level (HGB) of 72.0 g/L, and platelet count (PLT) of 16.0×10^9^/L. An abdominal ultrasound scan showed that the thickness of the spleen was approximately 3.2 cm. Contrast-enhanced CT scans of the whole abdomen indicated no signs of ovarian cancer recurrence. Peripheral blood smear examination revealed monocytosis with an increased monocyte percentage of 56.0% and naïve monocyte percentage of 15.0% in total nucleated cells. Morphological examination of a bone marrow (BM) aspirate revealed dysplasia in granulocytic and megakaryocytic cell lineages and an increased naïve monocyte percentage (approximately 10%) (**Figure 2A**). BM biopsy analysis revealed marked hypercellularity (approximately 90%) with prominent naïve monocytosis (**Figure S1**; **Supplemental Material**). Multiparametric flow cytometry analysis of BM aspirates showed that abnormal myeloid blasts with a percentage of 4.9% in total nucleated cells expressed the markers CD34, CD117, CD38, CD9^dim^, CD13^dim^, and CD33 and that naïve monocytes with a percentage of 8.4% in total nucleated cells expressed the markers CD64^bri^, CD11c^bri^, CD15^dim^, and CD14^neg^ (**Figure S2**; **Supplemental Material**). Cytogenetic analysis revealed an abnormal karyotype of “46, XX, t (11; 12)(p15; q13) [8]” (**Figure 2B**).

To clarify the molecular mechanism, comprehensive genetic testing was conducted (**Supplemental Material**). Targeted next-generation sequencing (NGS) of a panel of 80 genes was performed on her initial BM aspirate sample (Illumina NextSeq550 platform, amplicon library prep, amplicon mean coverage: 1813.4×, percent Q30 bases: 97.7%, uniformity of coverage: 97.8%) and identified *PTPN11* (T73I: 2.5%), *NRAS* (G12D: 3.2%, G12S: 7.7%, G13V: 22.4%), and *DNMT3A* (A571Lfs*80: 49.1%) somatic mutations. In addition, RNA sequencing (RNA-seq) identified a rare *NUP98-HOXC11* fusion. The breakpoints of *NUP98* and *HOXC11* were located in intron 12 or intron 1, respectively, resulting in a *NUP98-HOXC11* in-frame fusion transcript containing exon 12 of *NUP98* fused to exon 2 of *HOXC11*. To confirm the *NUP98-HOXC11* fusion, reverse transcription polymerase chain reaction (RT-PCR) was performed with a pair of primers, forward (at *NUP98* exon 12): 5’-TCTTGGTACAGGAGCCTTTGG-3’, and reverse (at *HOXC11* exon 2): 5’-GTTCCCGGATCTGGAATTTCG-3’, and then the amplified PCR products were purified to perform Sanger sequencing to verify the above finding (**Figure 2C**). Whole-exome sequencing (WES) of diagnostic BM samples identified four cancer susceptibility gene mutations associated with the *FA-BRCA* pathway, *RAD51C* (NM_058216.2: c.1027-1G>T), *BRIP1* (NM_032043.2: c.2393G>A, p. R798Q), *PALB2* (NM_024675.3: c.315G>C, p. E105D), and *FANCG* (NM_004629.1: c.1157C>G, p. P386R), which were further verified as heterozygous germline mutations by Sanger sequencing with buccal swab samples (**Figure S3**, **Supplemental Material**). *RAD51C* splicing mutation was predicted to be deleterious by the Human Splicing Finder and absent from the current Exome Aggregation Consortium (ExAC), 1000 Genomes Project (1000G), and Genome Aggregation Database (gnomAD), indicating that it may be a pathogenic/likely pathogenic variant. *BRIP1*, *PALB2*, and *FANCG* missense mutations were all rare in the current ExAC, 1000G, and gnomAD databases (Minor allele frequencies < 0.0001), and reported in ClinVar under “uncertain significance.” Furthermore, no other exonic variants were detected in genes associated with predisposition to cancer.

The patient’s past medical history provided the medical context that she had developed ovarian cancer with no history of cancer in her family in September 2016 (four years prior). Then she received six cycles of docetaxel and carboplatin (DC) combination chemotherapy [docetaxel, intravenous (I.V.) infusion by 60 minutes, 70 mg/m^2^, d1; carboplatin, I.V. infusion over 30 minutes, AUC (area under the plasma-concentration-versus-time curve) of 5 mg/mL/min, d1], followed by two cycles of docetaxel maintenance therapy (docetaxel, I.V. infusion by 60 minutes, 70 mg/m^2^, d1) after a definitive diagnosis from October 2016 to May 2017 in the medical oncology department of our hospital. After the primary treatment, she received a routine follow-up nearly every 3 months for the next 3 years. All the obtained results of the examination and laboratory tests were normal, and she had no signs or symptoms of ovarian cancer recurrence.

Based on the above results, the patient’s final diagnosis was t-CMML with the *NUP98-HOXC11* fusion and secondary to primary ovarian cancer with inherited cancer predisposition gene mutations of FA genes. Considering the extremely poor prognosis, two courses of a highly intensive induction chemotherapy regimen with DAC (decitabine, 20 mg/m^2^, d1-5) and HAA (homoharringtonine, 2 mg/m^2^, d1-7; cytarabine, 10 mg/m^2^, q12h, d1-10) were administered from 2 July 2020 to 20 August 2020. Subsequently, the patient presented with obvious pancytopenia and suffered from severe and persistent bone marrow suppression and delayed post−chemotherapy bone marrow recovery with a poor response to G-CSF treatment. On 2 September 2020, a CBC test indicated remaining pancytopenia with a WBC of 0.9×10^9^/L, HGB of 73.0 g/L, and PLT of 36.0×10^9^/L. Then, the patient asked to be discharged and transferred to her local hospital for personal reasons.

## Discussion

Fanconi anemia (FA) is a rare human genetic disease that occurs following germline mutations associated with the FA pathway and is clinically characterized by malformations, BMF, immune deficiency, and an extremely high predisposition to various cancers. The “FA pathway” is also known as the “*FA-BRCA* pathway” because many of the FA genes are related to breast cancer (BRCA) and/or ovarian cancer (7, 8). FA patients display a wide spectrum of diverse clinical manifestations; even so, only a small number of them present a typical phenotype, and one−third of individuals with FA have a normal appearance (6). Thus, it is a major challenge for clinicians to make an accurate diagnosis for FA patients. Chromosome breakage analysis may be a gold standard test, which is, nonetheless, not widely applied due to its false-negative traits and the inaccessibility in some poorly equipped hospitals (9, 10). According to previous literature, non-FA (or underlying FA) patients carrying germline FA gene mutations also present with an increased risk of developing BMF, immune deficiency, and cancer susceptibility under some circumstances (5, 11, 12).

Our patient had no signs or symptoms of developmental abnormalities or organ defects, and unfortunately, she did not receive a chromosome breakage test. Genetic testing indicated that she was born with four *FA-BRCA* pathway gene mutations in *RAD51C, BRIP1, PALB2*, and *FANCG*. Moreover, *RAD51C* (*RAD51* paralog C, alias *FANCO*), *BRIP1* (*BRCA*1−interacting protein carboxy−terminal helicase 1, alias *FANCJ*), and *PALB2* (partner and localizer of *BRCA2*, alias *FANCN*) have been fully implicated in an increased risk for breast and ovarian cancer (**Table 1**) (13–15). We hypothesize that the patient was possibly born with an increased predisposition to develop BMF, hematopoietic neoplasms, and breast and ovarian cancer due to a mild or moderate impaired *FA-BRCA* pathway.

**Table 1:** Association of defects in FA genes with predisposition to non-FA cancers.

References	Gene	Pathway	Function	Hematologic malignancy	Other malignancy
Meindl A et al. (13)	*RAD51C/FANCO*	Fanconi anemia/BRCA pathway	Regulate DNA repair by homologous recombination, and maintain genome stability	MDS, leukemia	Breast-ovarian cancer
Moyer CL et al. (14)	*BRIP1/FANCJ*			MDS, leukemia	Ovarian cancer, Breast cancer
Antoniou AC et al. (15)	*PALB2/FANCN*			MDS, leukemia	Breast cancer
Nepal M et al. (5)	*FANCG*			MDS, leukemia	.

FA, Fanconi anemia; MDS, Myelodysplastic Syndromes.

At the cellular level, her cells are hypersensitive to DNA-crosslinking agents, cytotoxic chemotherapeutics, and ionizing radiation due to corrupted DNA damage repair (3, 5). Over time, cumulative genomic instability renders cells malignant by clonal evolution and neoplastic transformation through uncontrolled proliferation, escape from programmed cell death, and evasion of immune surveillance. Consequently, she developed ovarian cancer at 50 years of age and then received six cycles of docetaxel and carboplatin (DC) combination chemotherapy followed by two cycles of docetaxel maintenance therapy. Cytotoxic chemotherapeutic exposure aggravated the tendency of hematopoietic stem progenitor cells to undergo malignant transformation and BMF. Three years later, she suffered from a secondary cancer of t-CMML and severe and persistent BMF after two courses of an induction chemotherapy regimen with DAC-HAA. The potential biological mechanisms behind the pathogenesis of t-CMML could be generally summarized as follows (1). Genomic instability due to the *RAD51C, BRIP1, PALB2*, and *FANCG* germline mutations of the FA pathway and long-term exposure to cytotoxic drugs aggravated the disease. (2). The selective pressure of cytotoxic drugs promoted the emergence of somatic variants (*DNMT3A*, *NRAS*, and *PTPN11* mutations) in subclone cells and led to the development of clonal hematopoiesis and evolution to hematopoietic malignancies (16, 17) (3). *NUP98-HOXC11* fusion also impaired hematopoietic stem cell self-renewal and caused malignant transformation directly (18) (**Figure 1C**).

t-CMML is a rare hematologic malignancy characterized by exposure to prior chemotherapy and/or radiation therapy used to treat a primary cancer (19). Takahashi K et al. (2013) performed the first systematic study to show the clinical characteristics and outcomes of t-CMML among a reasonably large number of 358 CMML patients (20). A total of 39 (11%) patients were defined as having t-CMML, and the median latency to develop t-CMML was 6 years (range: 1-32 years). The most common primary cancers in the t-CMML group were lymphoid malignancies (37%), followed by prostate cancer (31%) and breast or ovarian cancer (18%). They concluded that t-CMML was associated with higher-risk cytogenetics and manifests a poor prognosis. Subsequently, Subari S et al. (2015) and Patnaik MM et al. (2017) conducted two other large-scale studies of t-CMML in 265 and 497 CMML patients (21, 22), respectively, and they drew many conclusions similar to those of Takahashi K et al. To the best of our knowledge, no analysis of germline mutations in cancer susceptibility genes in t-CMML has been conducted, although systematic studies of the clinical characteristics and prognosis of the disease have been conducted. Thus, our case report may be the first relevant study.

*NUP98* fusion was first described by Nakamura T et al. and Borrow J et al. (1996) in AML patients with recurrent t (7;11) (p15; p15) translocation that resulted in a *NUP98-HOXA9* fusion gene (23, 24). Since then, it has been identified in a spectrum of hematologic malignancies, including AML, MDS, CML, T-ALL, and mixed-phenotype acute leukemia (MPAL), with a low incidence (<5%) (25). *NUP98* fusion can induce abnormal transcriptional or epigenetic regulation and drive leukemogenesis through complex molecular mechanisms (18). In particular, *NUP98* fusion is rarely reported in CMML patients, and we found only four relevant cases to date (**Table 2**). Currently, over 30 *NUP98* fusion partners have been identified, but the *HOXC11* partner is fairly rare. There are only four *NUP98-HOXC11* fusion cases reported by Taketani T et al. (2002) and Gu BW et al. (2003) (30, 31), which identified one case in a pediatric patient and three in adult patients with *de novo* AML. To the best of our knowledge, this is the first report identifying a rare *NUP98-HOXC11* fusion in a t-CMML patient.

**Table 2:** Summary of CMML cases with *NUP98* fusions reported in the literature.

References	Partners	Disease	Age	Sex	Karyotype	Primary cancer	Latency	Exposure
Hatano et al. (26)	*HOXA9*	CMML	45	M	46,XY,t(7;11)(p15;p15)	.	.	.
Wong et al. (27)	*HOXA9*	CMML	31	M	46, XY, t(7;11)(p15;p15)	.	.	.
Soler G et al. (28)	*AF10*	CMML	83	M	46, XY, t(10;11)(p12;p15)	.	.	.
Hayashi Y et al. (29)	*HBO1*	t-CMML	69	F	46,XX, t(11;17)(p11.5;q21)	Gastric cancer	3 year	fluorouracil,cisplatin,docetaxel,gemcitabine

CMML, chronic myelomonocytic leukemia; t-CMML, therapy-related chronic myelomonocytic leukemia; M, male; F, female.

In summary, we report the first case of a non-FA (or underlying FA) patient born with multiple FA gene mutations who developed ovarian cancer and secondary t-CMML with a rare *NUP98-HOXC11* fusion. Conventional chemotherapy and radiation therapy appear to be “double-edged swords”, as they kill cancer cells and induce secondary BMF, immune deficiency, and cancer (32). Particularly, it is difficult for clinicians to balance the clinical efficacy and adverse outcomes in cancer patients carrying cancer susceptibility gene mutations. From this case, we learn that cancer patients with high clinical suspicion for germline predisposition, such as secondary or early-onset hematologic malignancy patients, are desirable for appropriate genetic counseling and testing. Actually, those patients may benefit from timely diagnosis and personalized management, such as a better donor selection strategy, reduced-intensity conditioning for allogeneic hematopoietic stem cell transplantation, altered cytotoxic chemotherapeutics, and lifelong cancer surveillance.

## Data availability statement

The datasets presented in this study can be found in online repositories. The names of the repository/repositories and accession number(s) can be found below: https://www.ncbi.nlm.nih.gov/bioproject/PRJNA881251/.

## Ethics statement

The studies involving human participants were reviewed and approved by The Medical Ethics Committee of Tongji Hospital, Tongji Medical College, Huazhong University of Science and Technology. The patients/participants provided their written informed consent to participate in this study. Written informed consent was obtained from the individual(s) for the publication of any potentially identifiable images or data included in this article.

## Author contributions

KS collected and analyzed data, wrote the manuscript, and drew the figures. MZ performed RNAseq and analyzed data. JCW performed NGS and analyzed data. WM performed WES and analyzed data. JW performed morphological examination. CW performed flow cytometry and analyzed data. SX performed cytogenetic analysis. ZH and MX directed the research and reviewed the manuscript. All authors contributed to the article and approved the submitted version.

## Funding

This work was supported by the National Natural Science Foundation of China (No.81770211 to MX).

## Acknowledgments

The authors would like to thank the patient and her family for consenting to report this rare case, Perfectgen Diagnostics (Ezhou, Hubei Province) for providing DNA sequencing service, and all the faculty and staff in our Clinical and Laboratory Unit for clinical and technical support.

## Conflict of interest

The authors declare that the research was conducted in the absence of any commercial or financial relationships that could be construed as a potential conflict of interest.

## Publisher’s note

All claims expressed in this article are solely those of the authors and do not necessarily represent those of their affiliated organizations, or those of the publisher, the editors and the reviewers. Any product that may be evaluated in this article, or claim that may be made by its manufacturer, is not guaranteed or endorsed by the publisher.
