# Supplementary material for: Inherited heterozygous Fanconi anemia gene mutations in a therapy-related CMML patient with a rare NUP98-HOXC11 fusion: A case report
Source: Front Oncol. 2022 Oct 19;12:1036511. doi: 10.3389/fonc.2022.1036511 (PMC9626966; doi:10.3389/fonc.2022.1036511)

# *Supplementary Material*

## 1 Supplementary Data

Targeted next-generation sequencing (NGS) of a panel of 80 genes involved in myeloid neoplasms was performed on initial bone marrow (BM) aspirate sample. The library was prepared with AmpliSeq™ Library PLUS for Illumina, and paired-end sequencing was performed on NextSeq™ 550 platform (Illumina, San Diego, USA). The alignment and variant calling were performed using the DNA Amplicon workflow with default parameters on BaseSpace Sequence Hub (Illumina). Generated variants were further annotated using Annovar.

For whole-exome sequencing (WES) on diagnostic BM sample, the library was constructed by using Fast Library Prep Kit, and then the whole exons were captured with AIExome Enrichment Kit V1 (iGeneTech, Beijing, China). Sequencing was performed on Illumina platform (Illumina, San Diego, USA) with 150 base paired-end reads. Raw reads were filtered to remove low quality reads by using FastQC. Then clean reads were mapped to the reference genome GRCh37 by using BWA. After removing duplications, SNV and InDel were called and annotated by using GATK.

To search for the potential fusion gene, we used the patient's BM samples collected at diagnosis to perform RNA sequencing by Illumina platform (Illumina, San Diego, USA), and using STAR-Fusion (STAR Methods, https://github.com/alexdobin/STAR) for the fusion transcripts detection.

## 2 Supplementary Table

**Supplementary Table S1.** Gene list in targeted NGS of 80 genes, covering mutational hotspot regions or whole coding sequences (CDS) within 5 intronic base pairs around exons.

| **Gene list** | | | | | | |
|---|---|---|---|---|---|---|
| *ABL1* | *ALPP* | *ASXL1* | *ASXL2* | *BCL7A* | *BCOR* | *BCORL1* |
| *CALR* | *CBL* | *CBLB* | *CCND2* | *CDKN2A* | *CEBPA* | *CSF1R* |
| *CSF3R* | *CSNK1A1* | *CTCF* | *DNMT3A* | *ETNK1* | *ETV6* | *EZH2* |
| *FAM5C* | *FAT1* | *FBXW7* | *FGFR1* | *FGFR3* | *FLT3* | *FOXO1* |
| *GATA1* | *GATA2* | *GNAS* | *GNB1* | *HRAS* | *IDH1* | *IDH2* |
| *IKZF1* | *JAK1* | *JAK2* | *JAK3* | *KDM6A* | *KIT* | *KRAS* |
| *LTB* | *LUC7L2* | *MPL* | *NF1* | *NFE2* | *NPM1* | *NRAS* |
| *PDGFRA* | *PHF6* | *PIGA* | *PPM1D* | *PRPF40B* | *PTPN11* | *RAD21* |
| *RECQL4* | *RET* | *RIT1* | *RUNX1* | *SETBP1* | *SF1* | *SF3A1* |
| *SF3B1* | *SH2B3* | *SLC34A2* | *SMC1A* | *SMC3* | *SOCS1* | *SRSF2* |
| *STAG2* | *STAT3* | *STAT5B* | *TET1* | *TET2* | *TP53* | *U2AF1* |
| *U2AF2* | *WT1* | *ZRSR2* | | | | |

### 3. Supplementary Figures

**Supplementary Figure 1.**

**Figure S1.** BM biopsy revealed marked hypercellularity (~90%) with prominent naïve monocytosis. (Hematoxylin-eosin stained, A shows original magnification ×100, B shows original magnification ×400).

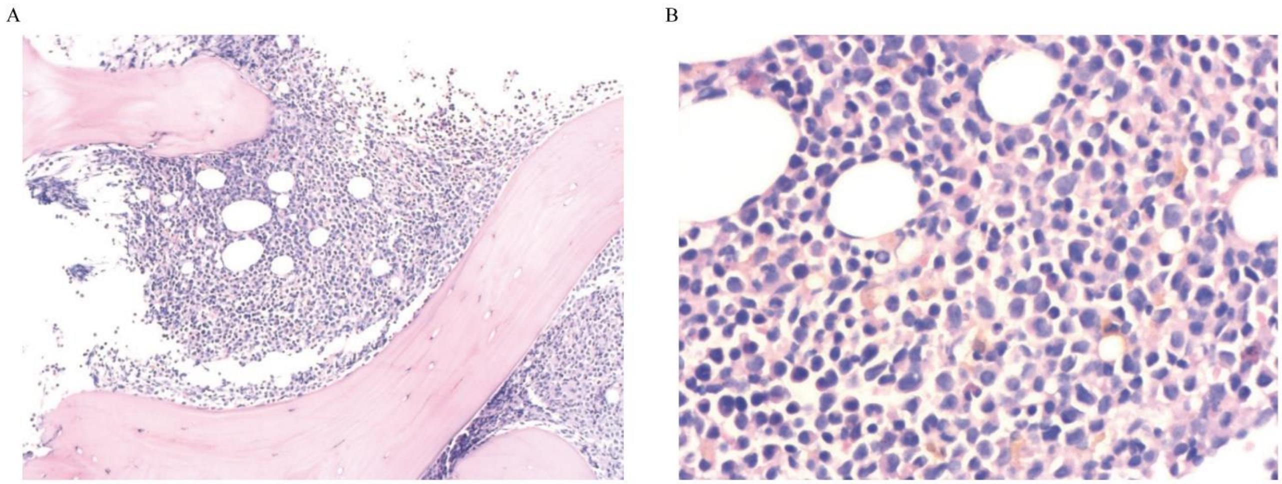

## Supplementary Figure 2.

**Figure S2.** Immunophenotype of the diagnostic BM sample.

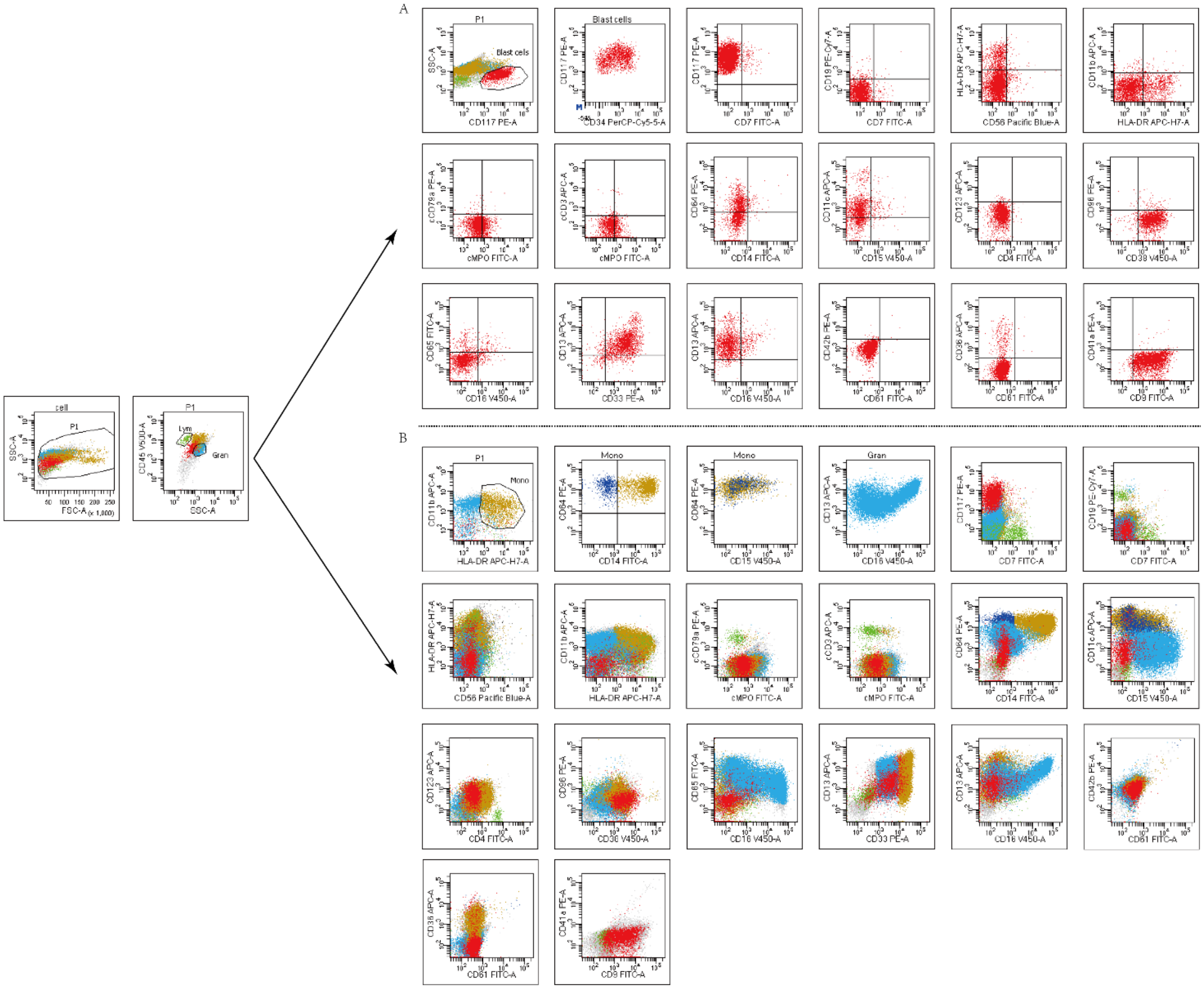

**Supplementary Figure 3.**

**Figure S3.** Germline confirmations were carried out with testing in the buccal swab sample by Sanger sequencing.

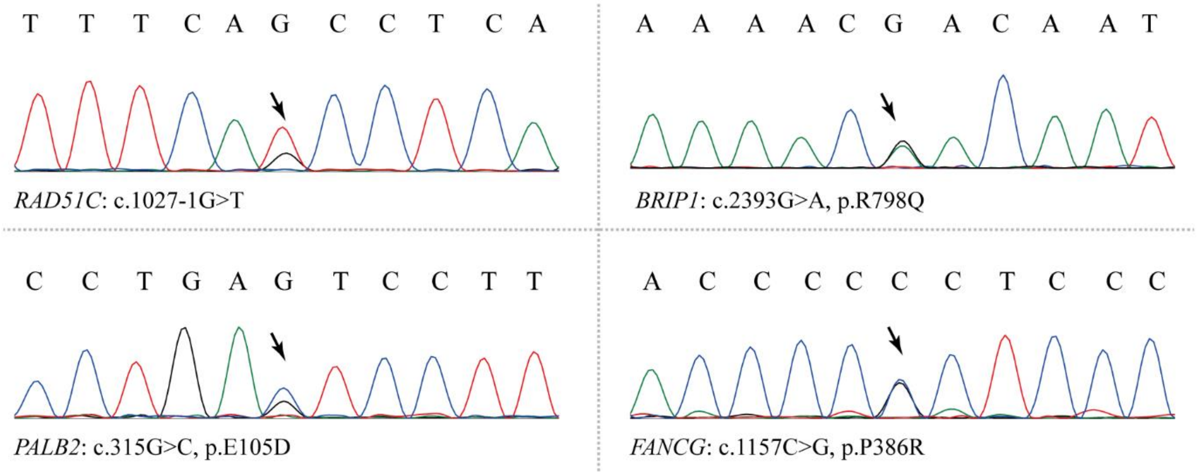

Supplement: Supplementary file 1 [file DataSheet_1.pdf]
